# Simultaneous evaluation of antioxidative serum profiles facilitates the diagnostic screening of autism spectrum disorder in under-6-year-old children

**DOI:** 10.1038/s41598-020-77328-z

**Published:** 2020-11-26

**Authors:** Aki Hirayama, Keisuke Wakusawa, Toru Fujioka, Keiko Iwata, Noriyoshi Usui, Daisuke Kurita, Yosuke Kameno, Tomoyasu Wakuda, Shu Takagai, Takaharu Hirai, Takahiro Nara, Hiromu Ito, Yumiko Nagano, Shigeru Oowada, Masatsugu Tsujii, Kenji J. Tsuchiya, Hideo Matsuzaki

**Affiliations:** 1 Center for Integrative Medicine, Tsukuba University of Technology, Tsukuba, Japan; 2 Department of Developmental Neuropsychiatry, Miyagi Children’s Hospital, Sendai, Japan; 3 Research Center for Child Mental Development, University of Fukui, Fukui, Japan; 4 United Graduate School of Child Development, Osaka University, Osaka, Japan; 5 Life Science Innovation Center, University of Fukui, Fukui, Japan; 6 Center for Medical Research and Education, Department of Neuroscience and Cell Biology, Graduate School of Medicine, Osaka University, Osaka, Japan; 7 Department of Neuroscience and Cell Biology, Graduate School of Medicine, Osaka University, Osaka, Japan; 8 Global Center for Medical Engineering and Informatics, Osaka University, Osaka, Japan; 9 Addiction Research Unit, Osaka Psychiatric Research Center, Osaka Psychiatric Medical Center, Osaka, Japan; 10 Department of Psychiatry, Hamamatsu University School of Medicine, Hamamatsu, Japan; 11 Department of Child and Adolescent Psychiatry, Hamamatsu University School of Medicine, Hamamatsu, Japan; 12 Department of Community Health Nursing, School of Medical Sciences, University of Fukui, Fukui, Japan; 13 Graduate School of Medical and Dental Sciences, Kagoshima University, Kagoshima, Japan; 14 Asao Clinic, Kawasaki, Japan; 15 School of Contemporary Sociology, Chukyo University, Toyota, Japan; 16 Research Center for Child Mental Development, Hamamatsu University School of Medicine, Hamamatsu, Japan

**Keywords:** Diagnostic markers, Autism spectrum disorders

## Abstract

This case–control study aimed to assess oxidative stress alterations in autism spectrum disorder (ASD). We used the MULTIS method, an electron spin resonance-based technique measuring multiple free radical scavenging activities simultaneously, in combination with conventional oxidative stress markers to investigate the ability of this MULTIS approach as a non-behavioural diagnostic tool for children with ASD. Serum samples of 39 children with ASD and 58 age-matched children with typical development were analysed. The ASD group showed decreased hydroxyl radical (^·^OH) and singlet oxygen scavenging activity with increased serum coenzyme Q10 oxidation rate, indicating a prooxidative tendency in ASD. By contrast, scavenging activities against superoxide (O_2_^·−^) and alkoxyl radical (RO^·^) were increased in the ASD group suggesting antioxidative shifts. In the subgroup analysis of 6-year-olds or younger, the combination of ^·^OH, O_2_^·−^, and RO^·^ scavenging activities predicted ASD with high odds ratio (50.4), positive likelihood (12.6), and percentage of correct classification (87.0%). Our results indicate that oxidative stress in children with ASD is not simply elevated but rather shows a compensatory shift. MULTIS measurements may serve as a very powerful non-behavioural tool for the diagnosis of ASD in children.

## Introduction

Autism spectrum disorder (ASD) is a neurodevelopmental disorder characterized by pervasive abnormalities in social interaction and communication, repetitive behaviours, and restricted interests. In most cases, the specific underlying pathophysiology of this disorder remains poorly understood^1–3^. Though multiple theories have been proposed about the aetiology of ASD, it is still difficult to identify a specific aetiology or developmental pathway that connects to the clinical phenotype to ASD^4^. Despite new knowledge and theories regarding ASD biology, current research methods are not able to provide individualized information^5^.

The aetiology of ASD is thought to involve complex, multigenic interactions and possible environmental contributions^6^. A number of risk factors, including genetic, infectious, metabolic, nutritional, and environmental factors, have been investigated, although specific causes are detected in less than 10–12% of cases^7^. Genetic and environmental factors that induce inflammation and immune responses in the brain are thought to be involved in the pathogenesis of ASD and have been extensively studied in recent years^8–10^. Prenatal and perinatal exposures to a certain circumstance are considered as a risk factor for ASD^10–13^.

There is strong evidence that early diagnosis and treatment can make a major difference in the outcome for many children with ASD and related conditions^14^. Although the global prevalence of ASD in children has increased substantially over recent decades^15–17^, effective treatments are lacking. Therapeutic interventions are most effective if started early in life. On the other hand, the diagnosis of ASD is sometimes left behind because it is based on the identification of autistic behaviour that may not appear until the disorder is well established^18^. Biomarkers are objective measurement tools for pathophysiological or biological processes or pharmacologic responses to therapeutic interventions^19^. Biomarkers that help screen children at risk during the pre-symptomatic period are useful for early diagnosis, patient stratification, and prediction of treatment response^18^. However, there are no blood-based diagnostic tools, nor is there sufficient evidence regarding the efficacy of specific pharmacological treatments for the core symptoms of ASD^4,20–22^. As the Research Domain Criteria developed by the National Institute of Mental Health divides human mental functioning into five specific domains including specific behavioural or emotional components^23^, we may use biomarkers effectively in an integrated way and utilize them in our clinical activities in the future. As ASD symptoms are mapped onto specific biological processes and its biomarkers, further insights into disease processes and potential therapeutics are expected to emerge. Therefore, reliable ASD biomarkers to assist in the diagnosis or treatment monitoring of this disorder are urgently needed.

Oxidative stress is a pathophysiological condition currently recognized as one possible mechanism underlying ASD, among various other diseases^24–26^. A number of investigations attempted to apply oxidative stress markers as an early diagnosis tool for ASD. A recent meta-analysis revealed that children with ASD show decreased glutathione peroxidase (GPx) activity and decreased plasma levels of methionine, cysteine, and glutathione of the reduced form (GSH) along with elevated oxidized glutathione levels^25^. However, the results for other oxidative stress markers and antioxidants, including coenzyme Q10 (CoQ10), tocopherols, and superoxide dismutase (SOD), are highly variable, and a comprehensive explanation of all of these oxidative stress-related reactions has not been obtained^10,25,27,28^.

So far, investigations of oxidative stress have mainly focused on cellular responses invoked by oxidative stimuli, including NF-E2-related factor 2 (Nrf2)-Kelch-like ECH-associated protein 1 (Keap1) and nuclear factor κ-light-chain-enhancer of activated B cells (NF-κB) pathways^29–31^. Sulforaphane, an isothiocyanate with indirect antioxidant effects derived from broccoli sprouts and seeds, was recently shown to lead to improvements in the behaviour and social responsiveness of children with ASD^32^. One of the most important sulforaphane targets is Nrf2, widely known for its ability to regulate the expression of cytoprotective enzymes with antioxidative, prosurvival, and detoxification effects^33^.

Nevertheless, the stimulatory aspect of oxidative stress evoking subsequent responses has not been clarified yet. Due to their high reaction rates, the identity of reactive oxygen species (ROS) that act as stimulators of oxidative stress reaction mechanisms remains unclear in many diseases^34,35^. Until now, it has been very difficult to fully characterize oxidative stress reactions occurring in vivo. For this purpose, we developed an electron spin resonance (ESR)-based analytical method for assessing multiple ROS scavenging activities, namely hydroxyl radical (^·^OH), alkoxyl radical (RO^·^, t-BuO^·^), peroxyl radical (ROO^·^, t-BuOO^·^), superoxide (O_2_^·−^), and singlet oxygen (^1^O_2_), in biological samples (MULTIS method)^36–39^. This method allows the simultaneous evaluation of multiple antioxidative systems, i.e., scavenging of hydroxyl radicals by GSH and superoxide by the superoxide dismutase system, and the detailed characterization of oxidative stress-related reactions. Using this MULTIS method in various clinical samples and experimental models, we have already provided evidence that oxidative stress is not simply increased in vivo but shows complicated alterations involving multiple ROS and antioxidants^36,37,39^.

In the present study, we first aimed to comprehensively describe the oxidative stress and antioxidative system alterations in children with ASD by using the MULTIS method combined with conventional oxidative stress markers. Moreover, based on these results, we also investigated the ability of MULTIS measurements as a non-behavioural diagnostic tool for ASD in children. Here, we also focused on whether this technique would help in the early diagnosis of preschool children with ASD under 6 years old. Early detection is essential for early intervention for ASD^21^, and the latter is a key issue in public health policy for ASD^40^. There has been an increasing number of published trials on psychosocial intervention programmes for preschool children with ASD^41^. However, as already described, many biomarkers are still preliminary for early diagnosis of ASD and need to be validated^18^. Our results strongly suggest that in addition to elevated levels of oxidative stress, complicated shifts of the oxidative-antioxidative balance are key factors in the pathological mechanisms underlying ASD, and this property can be applied to the diagnostic screening for ASD.

## Results

### Characteristics of the study population

Serum samples of 39 children with ASD and 58 age-matched healthy control children with typical development (TD) were analysed in this study. Among them, 19 in the ASD group and 26 in the TD group were 6 years old or younger. The profiles of both groups and results of the psychological measurements of children with ASD are shown in Table 1. ASD is known to be more common in males^42^, and there was a significant gender difference between the ASD and TD groups in the current study. Thus, we also analysed the group restricted to male children. There were no significant intergroup differences in age, weight, height, and body mass index. The developmental quotient (DQ) was significantly lower in children with ASD.

**Table 1:** Patients profiles of the ASD and TD groups.

	Total			6 years old and under		
	ASD	TD	*P*	ASD	TD	*P*
	Mean/median (95% CI)	Mean/median (95% CI)		Mean/median (95% CI)	Mean/median (95% CI)	
Age (y.o.)	7.7/6.5 (6.2–9.2)	7.3/6.6 (6.4–8.3)	0.960	3.8/4.0 (3.3–4.1)	4.0/4.1 (3.5–4.4)	0.572
M/F	32/7	30/28	0.004	14/5	14/12	0.175
Weight (kg)	26.8/23.8 (22.4–31.2)	25.3/20.9 (22.1–28.5)	0.691	16.2/15.6 (14.6–17.7)	15.6/15.5 (14.6–16.7)	0.695
Height (cm)	121.3/123.5 (112.8–129.8)	119.6/116.1 (113.7–125.5)	0.923	97.9/98.7 (94.1–101.6)	99.5/98.3 (96.2–102.8)	0.491
BMI	17.1/16.5 (16.4–17.9)	16.6/16.1 (16.1–17.2)	0.127	16.8/16.0 (15.8–17.8)	15.7/15.4 (15.3–16.2)	0.100
IQ/DQ	78/72 (68–87)	104/103 (101–107)	< 0.001	60/57 (52–69)	103/102 (98–107)	< 0.001
ADI-A	19/20 (16–21)	NA		18/18 (14–22)	NA	
ADI-B	12/12 (11–13)	NA		10/12 (9–12)	NA	
ADI-C	3.8/4.0 (3.0–4.5)	NA		3.2/3.0 (2.3–4.1)	NA	

All indices are expressed as the mean/median.

*ASD* autism spectrum disorder, *TD* typical development, *CI* confidence interval, *M/F* male-to-female ratio, *BMI* body mass index, *IQ* intelligence quotient, *DQ* developmental quotient, *ADI* Autism Diagnostic Interview-Revised, *ADI-A* ADI-R domain A score, *ADI-B* ADI-R domain B score, *ADI-C* ADI-R domain C score.

### Children with ASD showed decreased hydroxyl radical scavenging activity and increased superoxide and alkoxyl radical scavenging activities

The obtained ESR spectra of the spin adducts for hydroxyl radical (^·^OH), alkoxyl radical (RO^·^), alkylperoxyl radical (ROO^·^), superoxide (O_2_^·−^), and singlet oxygen (^1^O_2_) agreed with previously reported spectra of the corresponding radical adducts, confirmed by their hyperfine coupling constants^37^. The addition of sera decreased the signal amplitude, and the scavenging activity was calculated according to the previously described method.

**Figure 1:**
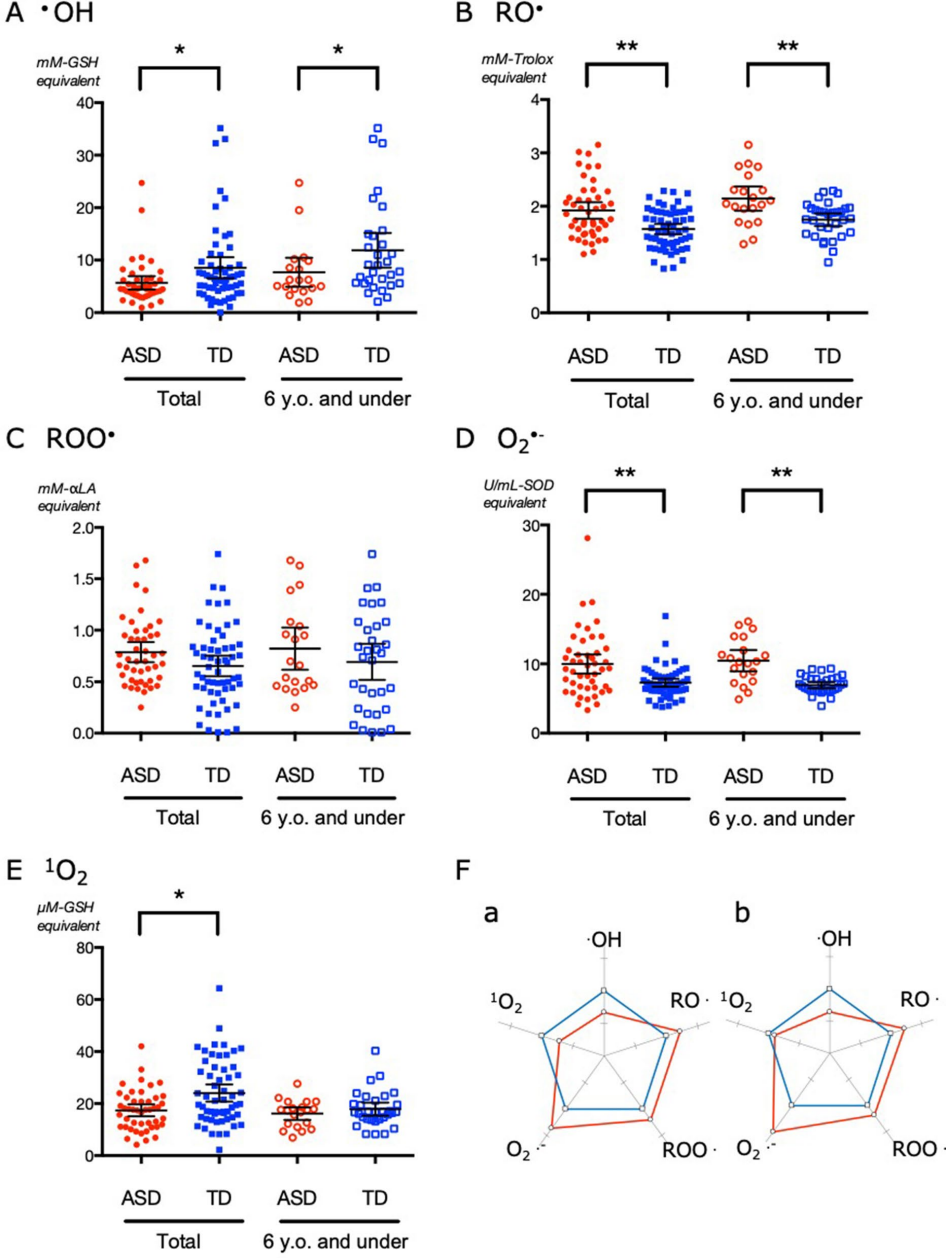
Serum antioxidative profiles against multiple reactive oxygen species (ROS) in the autism spectrum disorder (ASD) and typical development (TD) groups. The targeted ROS are ^·^OH (**A**), RO^·^ (**B**), ROO^·^ (**C**), O_2_^·−^ (**D**), and ^1^O_2_ (**E**). The scavenging activities are converted into equivalent units of specific scavengers shown in Table 1. “6 y.o. and under” indicates the results in children 6 years old or younger in each group. Lines represent means ± 95% confidence intervals. *P < 0.05, **P < 0.01 between the two groups. (**F**) Radar charts of serum scavenging activities comparing the ASD and TD groups. (**Fa**) All participating children. (**Fb**) Children aged 6 years or younger. Percent differences in the scavenging activities in the ASD group (red) are shown with respect to those in the TD group (blue).

The multiple radical scavenging activities in both ASD and TD children are shown in Fig. 1 and Table 2. The ^·^OH scavenging activity in the ASD group was significantly lower than that in the TD group. By contrast, the ASD group showed significantly increased scavenging activities against O_2_^·−^ and RO^·^ in comparison to the TD group, indicating that redox changes in the ASD group do not simply represent enhanced oxidative stress but more complicated antioxidative shifts. There were no significant differences in the scavenging activities against ROO^·^. The ^1^O_2_ scavenging activity of the ASD group was significantly lower than that of the TD group. However, this tendency was not statistically significant in the cohort of children with an age of 6 years or less. A radar chart illustrating the antioxidative profile against multiple ROS in the ASD group with respect to that in the TD group is shown in Fig. 1F. Children with ASD show a clear shift in the antioxidative profile towards decreased hydroxyl radical scavenging activity and increased alkoxyl radical and superoxide scavenging activities.

**Table 2:** Serum antioxidative profiles against multiple ROS in the ASD and TD groups.

ROS	Equivalent	Total			6 years old and under		
		ASD	TD	*P*	ASD	TD	*P*
		Mean (95% CI)	Mean (95% CI)		Mean (95% CI)	Mean (95% CI)	
Hydroxyl radical (^·^OH)	*mM-GSH*	5.9 (4.5–7.3)	8.5 (6.5–10.5)	0.034	7.7 (4.9–10.4)	12.5 (8.6–16.4)	0.036
Alkoxyl radical (RO^·^)	*mM-Trolox*	2.0 (1.8–2.2)	1.6 (1.5–1.7)	< 0.001	2.2 (1.9–2.4)	1.7 (1.6–1.9)	0.002
Alkyleroxyl radical (ROO^·^)	*mΜ-αLA*	0.8 (0.7–0.9)	0.7 (0.6–0.8)	0.094	0.8 (0.6–1.0)	0.7 (0.5–0.9)	0.215
Superoxide (O_2_^·−^)	*U/mL-SOD*	10.4 (8.9–11.9)	7.3 (6.7–7.9)	< 0.001	10.7 (9.2–12.0)	7.0 (6.5–7.6)	< 0.001
Singlet oxygen (^1^O_2_)	*mM-GSH*	17 (15–20)	24 (21–27)	0.016	16 (13–19)	18 (15–21)	0.634

ROS	Equivalent	Male			Male 6 years old and under		
		ASD	TD	*P*	ASD	TD	*P*
		Mean (95% CI)	Mean (95% CI)		Mean (95% CI)	Mean (95% CI)	
Hydroxyl radical (^·^OH)	*mM-GSH*	5.8 (4.1–7.5)	8.3 (5.8–10.7)	0.011	7.9 (4.0–11.6)	11.5 (6.7–16.3)	0.083
Alkoxyl radical (RO^·^)	*mM-Trolox*	1.9 (1.7–2.1)	1.6 (1.4–1.7)	0.006	2.2 (1.9–2.4)	1.7 (1.5–2.0)	0.019
Alkyleroxyl radical (ROO^·^)	*mΜ-αLA*	0.8 (0.7–0.9)	0.6 (0.5–0.8)	0.129	0.9 (0.6–1.1)	0.6 (0.2–0.9)	0.060
Superoxide (O_2_^·−^)	*U/mL-SOD*	10.6 (9.0–12.1)	7.5 (6.8–8.3)	0.001	11.1 (9.2–13.0)	7.5 (6.9–8.0)	< 0.001
Singlet oxygen (^1^O_2_)	*mM-GSH*	18 (15–20)	25 (20–30)	0.043	16 (12–19)	19 (14–24)	0.454

*ROS* reactive oxygen species, *ASD* autism spectrum disorder, *TD* typical development, *CI* confidence interval, *Trolox* 6-hydroxy-2,5,7,8-tetramethylchroman-2-carboxylic acid, *αLA* α-lipoic acid, *GSH* glutathione, *SOD* superoxide dismutase.

The results of the analysis with male children were mostly similar to those involving children of both sexes (Table 2). The only difference was that the difference in hydroxyl radical scavenging activity was no longer significant in the children aged 6 years or less.

### In the ASD group, both oxidative stress markers and antioxidants were increased

**Figure 2:**
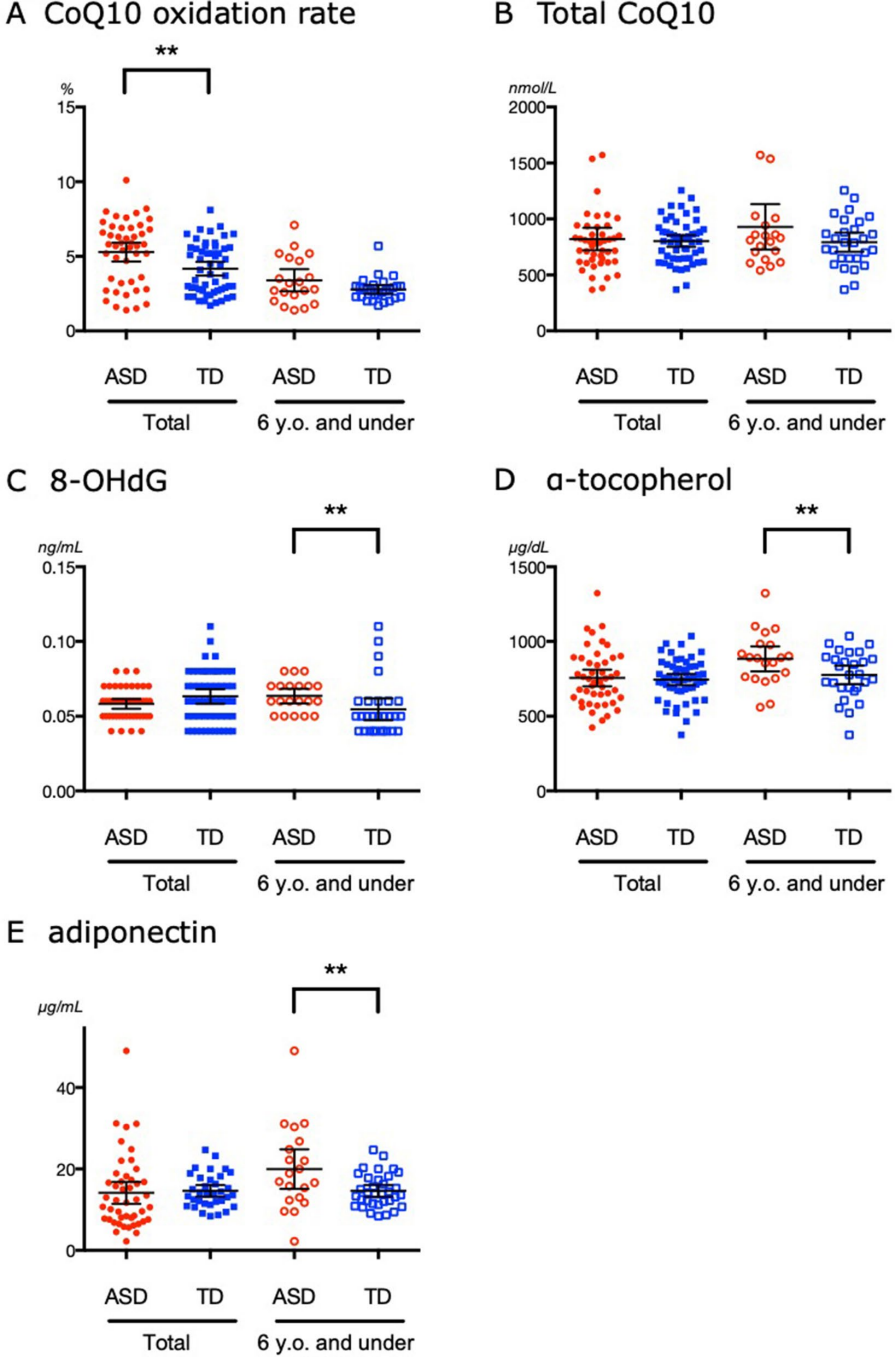
Oxidative stress markers and antioxidants in the autism spectrum disorder (ASD) and typical development (TD) groups. Panels (**A**), (**B**), (**C**), (**D**) and (**E**) show the coenzyme Q10 (CoQ10) oxidation ratio, total serum CoQ10, 8-hydroxy-2′-deoxyguanosine (8-OHdG), α-tocopherol, and adiponectin values, respectively. Lines represent means ± 95% confidence intervals. *P < 0.05, **P < 0.01 between the two groups.

**Figure 3:**
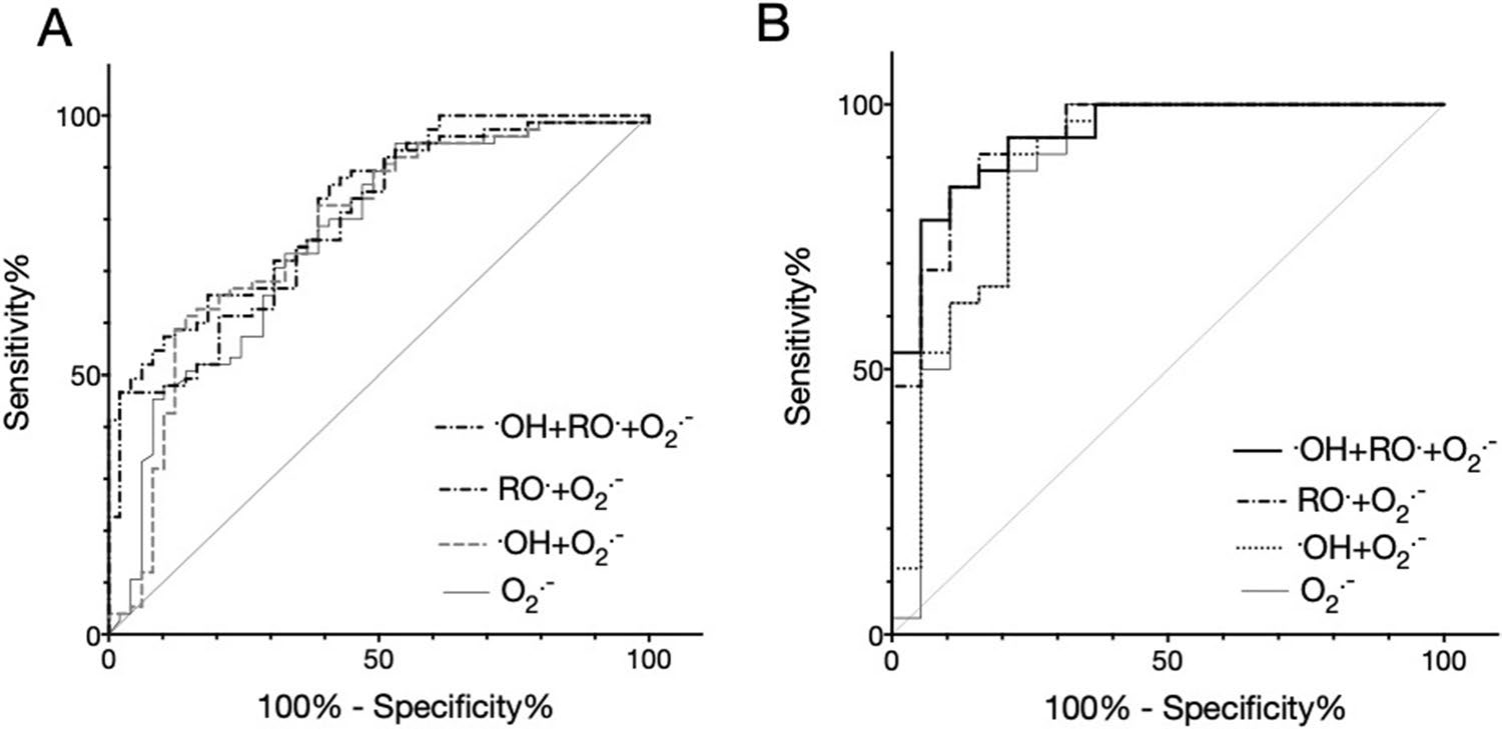
Receiver operating characteristic curves of the MULTIS method for autism spectrum disorder screening. The results of ^·^OH, RO^·^, and O_2_^·−^ scavenging activities and their combinations are shown. (**A**) Data from all participating children. (**B**) Data from children aged 6 years or younger.

The results regarding conventional oxidative stress markers and antioxidants are shown in Figs. 2 and 3 and Table 3. The CoQ10 oxidation rates in the ASD group and serum 8-hydroxy-2′-deoxyguanosine (8-OHdG) values in the ASD group of 6-year-olds or younger were significantly higher than those in the corresponding TD group, suggesting an increase in oxidative stress. However, the difference in antioxidants was noticeable in the group below 6 years of age. Significantly higher adiponectin values were only found in children with ASD who were 6 years old or younger.

**Table 3:** Oxidative stress markers and antioxidants in the ASD and TD groups.

Markers	Total			6 years old and under		
	ASD	TD	*P*	ASD	TD	*P*
	Mean (95% CI)	Mean (95% CI)		Mean (95% CI)	Mean (95% CI)	
CoQ10 Oxidation Rate (%)	5.2 (4.5–5.9)	4.2 (3.7–4.6)	0.012	3.5 (2.7–4.2)	2.8 (2.4–3.1)	0.244
Total CoQ10 (nmol/L)	829 (714–944)	802 (750–854)	0.552	937 (722–1151)	806 (703–910)	0.727
8-OHdG (pg/mL)	5.9 (5.6–6.2)	6.3 (5.8–6.8)	0.300	6.3 (5.8–6.8)	5.2 (4.4–6.0)	0.001
α-tocopherol (μg/dL)	762 (702–822)	744 (707–781)	0.967	878 (790–996)	780 (707–853)	0.119
Adiponectin (μg/mL)	14.8 (11.7–17.9)	14.6 (13.2–16.1)	0.398	19.8 (14.7–25.0)	15.5 (13.8–17.1)	0.156

Markers	Male			Male 6 years old and under		
	ASD	TD	*P*	ASD	TD	*P*
	Mean (95% CI)	Mean (95% CI)		Mean (95% CI)	Mean (95% CI)	
CoQ10 Oxidation Rate (%)	5.5 (4.8–6.2)	4.1 (3.4–4.7)	0.004	3.3 (2.3–4.2)	3.0 (2.4–3.6)	0.952
Total CoQ10 (nmol/L)	827 (711–942)	830 (753–906)	0.370	1003 (713–1293)	800 (659–941)	0.571
8-OHdG (pg/mL)	5.9 (5.5–6.2)	6.2 (5.5–6.9)	0.494	6.1 (5.5–6.7)	5.1 (4.2–6.0)	0.011
α-tocopherol (μg/dL)	730 (674–785)	742 (691–793)	0.594	857 (761–953)	774 (678–870)	0.174
Adiponectin (μg/mL)	13.9 (10.6–17.1)	13.9 (12.1–15.6)	0.129	22.3 (15.7–28.9)	14.9 (12.7–17.0)	0.030

*ASD* autism spectrum disorder, *TD* typical development, *CI* confidence interval, *CoQ10* coenzyme Q10, *8-OHdG* 8-hydroxy-2′-deoxyguanosine.

Again, the results of the analysis with male children were mostly similar to those of both sexes’ (Table 3). For 8-OHdG, there was a significant increase in ASD children with an age of 6 years or less compared to that in the age-matched male TD group.

### Sensitivity, specificity, and positive likelihood ratio of MULTIS for ASD diagnosis

To apply these ROS scavenging activity measurements in clinical screening, we established a prediction model for the probability of ASD using discriminant analysis (Table 4 and Fig. 3). Because ROS scavenging activities against ^·^OH, RO^·^, O_2_^·−^, and ^1^O_2_ showed significant differences between ASD and TD groups, we included these parameters as variables. In the group of male children aged 6 years or less, there was no significant difference in ^·^OH scavenging activity; thus, this parameter was excluded from the analysis.

**Table 4:** ASD screening accuracy of ROS scavenging activities alone and in combination.

ROS scavenging activity	Total						6 years old and younger					
	OR	Sens	Spec	LR+	CC	AUC	OR	Sens	Spec	LR+	CC	AUC
^·^OH	1.85	0.74	0.40	1.22	0.53	0.61	2.18	0.74	0.44	1.31	0.55	0.65
RO^·^	1.90	0.53	0.63	1.42	0.59	0.65	8.40	0.74	0.75	2.95	0.75	0.77
O_2_^·−^	4.82	0.69	0.68	2.17	0.69	0.76	27.10	0.74	0.91	7.86	0.84	0.87
^·^OH + RO^·^	2.51	0.57	0.65	1.65	0.62	0.69	6.64	0.72	0.72	2.57	0.72	0.79
^·^OH + O_2_^·−^	4.82	0.69	0.68	2.17	0.69	0.77	35.00	0.78	0.91	8.56	0.84	0.88
RO^·.^ + O_2_^·−^	4.13	0.67	0.67	2.02	0.71	0.79	32.50	0.68	0.94	10.90	0.84	0.94
^·^OH + RO^·.^ + O_2_^·−^	6.92	0.67	0.78	2.97	0.73	0.83	50.40	0.77	0.94	12.60	0.87	0.94
^·^OH + RO^·.^ + O_2_^·−^ + ^1^O_2_	8.86	0.76	0.74	2.92	0.75	0.85	31.40	0.68	0.94	10.60	0.84	0.95

ROS scavenging activity	Male						Male 6 years old and younger					
	OR	Sens	Spec	LR+	CC	AUC	OR	Sens	Spec	LR+	CC	AUC
^·^OH	3.4	0.84	0.40	1.4	0.64	0.70	1.6	0.71	0.39	1.2	0.53	0.65^b^
RO^·^	2.3	0.57	0.63	1.6	0.60	0.69	9.4	0.86	0.61	2.2	0.72	0.78
O_2_^·−^	9.5	0.60	0.87	4.5	0.72	0.72	20.0	0.71	0.89	6.4	0.81	0.88
^·^OH + RO^·^	4.9	0.68	0.70	2.3	0.69	0.77	7.3	0.79	0.67	2.4	0.72	0.82^b^
^·^OH + O_2_^·−^	6.1	0.65	0.77	2.8	0.72	0.77	20.0	0.71	0.89	6.4	0.82	0.88^b^
RO^·.^ + O_2_^·−^	6.9	0.68	0.77	2.9	0.72	0.79	42.5	0.71	0.94	12.9	0.88	0.94
^·^OH + RO^·.^ + O_2_^·−^	6.3	0.73	0.70	2.4	0.72	0.85	86.3	0.71	1.00	^a^	0.88	0.94^b^
^·^OH + RO^·.^ + O_2_^·−^ + ^1^O_2_	12.4	0.76	0.80	3.8	0.78	0.87	42.5	0.71	0.94	12.9	0.89	0.95^b^

*ROS* reactive oxygen species, *ASD* autism spectrum disorder, ^*·*^*OH* hydroxyl radical, *RO*^*·*^ alkoxyl radical, *O*_*2*_^*·−*^ superoxide, ^*1*^*O*_*2*_ singlet oxygen, *OR* odds ratio, *Sens* sensitivity, *Spec* specificity, *LR*+ positive likelihood ratio, *CC* percentage of correct classification, *AUC* area under the receiver operating characteristic curve.

^a^Incomputable because the specificity is 1.0

^b^Not significant.

Generally, the O_2_^·−^ scavenging activity showed the highest odds ratio (OR), sensitivity, specificity, positive likelihood (LR+), and percentage of correct classification (CC). Thus, these parameters showed higher values in combinations containing the variable O_2_^·−^. The discrimination of ASD based on ROS scavenging activities was markedly more functional in the group under 6 years of age. When ^·^OH, RO^·^, and O_2_^·−^ were employed, the LR+ increased to 12.8 with high values of CC (86.0%), OR (50.4), and high accuracy of the area under the receiver operating characteristic curve (AUC; 0.94). Analyses with two or more combinations of ROS met the generally accepted standards for the goodness of model fitting in multivariate analysis (AUC > 0.7). There were no significant correlations between discriminant and ADI-R scores (data not shown).

## Discussion

The involvement of oxidative stress in the pathophysiology of ASD has been reported frequently in the past decade and is now considered to be a major factor thereof. Previous reviews seem to have almost established that the antioxidative system involving GSH is attenuated in ASD patients^10,25,43^. However, there are no widely accepted theories for other oxidants and antioxidants, including SOD, CoQ10, ceruloplasmin, catalase, vitamins A, B, C, and D, as well as 2-thiobarbituric acid-reactive substances. Endres et al. tried to confirm their working hypothesis regarding decreased GSH concentrations in ASD but failed^44^.

Most ROS are based on superoxide originating from multiple systems, including NADPH oxidases, xanthine oxidase, and mitochondria. Superoxide is converted into hydroxyl radical in the presence of Fe^2+^. Hydroxyl radicals promote DNA damage in nuclei and lipid peroxidation in cellular membranes leading to the generation of lipid alkoxyl and alkylperoxyl radicals and further ROS chain reactions^45,46^. These chain reactions are mainly terminated by eliminating lipid peroxides by tocopherols and conversion to lipid alcohols.

Our results showed a decrease in hydroxyl radical and singlet oxygen scavenging activities along with elevated levels of the CoQ10 oxidation rate, except in the analysis limited to male children 6 years old or under. Because GSH is a major antioxidant against hydroxyl radicals and because of the almost established decrease in GSH and GPx activity in children with ASD^10,25,43^, it can be safely assumed that the hydroxyl radical elimination pathway is severely attenuated in ASD patients, and this is probably the primary and key mechanism for the oxidative damage in ASD.

**Figure 4:**
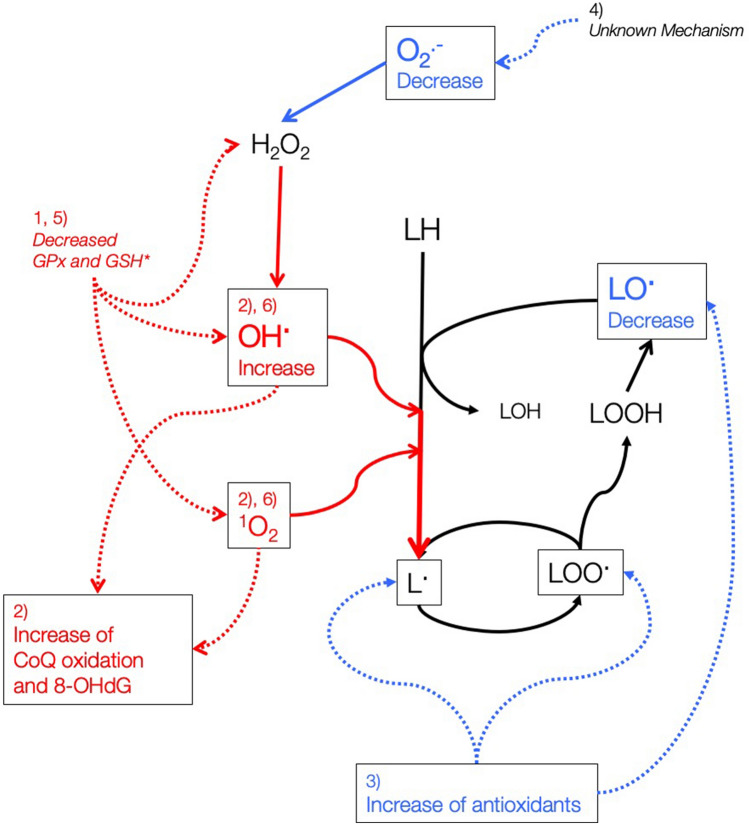
Proposed shifts in oxidative-antioxidative balances in children with autism spectrum disorder. The numbers indicated in the figure correspond to the following steps: (1) the primary pathogenesis is an attenuation of the reduced glutathione (GSH) system in the central nervous system^25^. (2) This increases the hydroxyl radical formation, leading to elevations in 8-hydroxy-2′-deoxyguanosine (8-OHdG) and coenzyme Q10 (CoQ10) oxidation rate^47^. (3) Step 2 may result in increased levels of lipid alkoxyl and alkylperoxyl radicals; however, the antioxidative activities against these radicals are enhanced by elevated α-tocopherol levels and unknown feedback system(s). (4) The superoxide scavenging activity is also increased via an unknown feedback system involving superoxide dismutase. (5) Despite the increase in superoxide dismutase activity, the cytoplasmic glutathione peroxidase (GPx) activity is reduced, so hydrogen peroxide (H_2_O_2_) may be present in relative excess. (6) This increase in hydrogen peroxide further enhances the production of hydroxyl radicals, and finally, this reactive oxygen species chain reaction expands. Molecules in squares indicate those evaluated in the present study. Asterisks indicate results reported in previous studies. Red letters and lines indicate prooxidative pathways, blue letters and lines indicate antioxidative pathways, and black letters and lines indicate unaffected or neutral pathways.

Interestingly, in contrast to the hydroxyl radical findings, the scavenging activities against superoxide and alkoxyl radicals were increased in ASD, and the serum levels of adiponectin were also increased in ASD children 6 years old or younger, suggesting an enforcement of the antioxidative and anti-inflammatory system. Therefore, our results indicate that the oxidative stress in ASD patients is not simply increased but shows a complicated imbalance, including multiple types of ROS, subsequent ROS chain reactions, and wide modifications in the antioxidant system. This imbalance may strongly influence the cerebral nervous system in early childhood that is susceptible to oxidative damage. Figure 4 displays our current hypothesis of the oxidative-antioxidative system in children with ASD derived from our present results in combination with findings in previous reports^25,47^. Meanwhile, there are no significant differences in ^·^OH scavenging activity in the subanalysis of male children aged 6 years or less. As mentioned above, there is a significant gender difference in the overall analysis, reflecting the prevalence of ASD in males. This difference could be a confounding factor in our analysis, and it is impossible to strictly exclude the possibility that the changes in oxidative parameters attributed to ASD are potentially brought about by the sex difference. However, the other ROS scavenging activities, conventional oxidative stress markers and antioxidants showed the same trend in the groups, including both sexes as in the male-only group, suggesting that the results in the former group may reflect the overall trend, with fewer factors attributable to sex differences.

In contrast to our findings, previous reports have uniformly shown that plasma/serum vitamin E levels are decreased in ASD patients. The reason for this discrepancy is unknown at present, but one possible factor is the age of the study participants, which is lower in the present study than in previous reports. The plasma concentration of α-tocopherol in humans is lowest in the neonatal period and increases with age^48,49^. It is important to note that both ASD and TD groups showed differentiation almost within the range of previously reported reference values of α-tocopherol^48^. α-Tocopherol is an essential vitamin that is taken up by the α-tocopherol transfer protein. Its levels are easily decreased due to a lack of intake, but pathological conditions with high α-tocopherol levels are very rare with a regular diet. In adults, over-supplementation of vitamin E is known to result in many adverse events, including carcinogenesis^50^. Together with the elevated adiponectin levels and increased serum superoxide scavenging activity, our results suggest that excessive antioxidation may have harmful effects in children with ASD. The brain tissue of human infants is extremely vulnerable to oxidative stress, but the details in individuals with ASD are unknown because there are currently no clinical ASD studies that investigate the function of mitochondria or ROS at the level of the synapse^51^. The hypothetical pathology presented in this study (Fig. 4) is highly probable to affect neuronal development.

The increases in SOD and adiponectin levels in our results suggest an adaptive mechanism that enhances the antioxidative system in ASD. The underlying mechanism of this adaption is not clear, although the Nrf2-Keap1 system is a strong candidate. The Nrf2-Keap1 system is well-known as a biological response system induced by oxidative stress^29,30^ and induces antioxidant enzymes, including glutathione-S-transferase and γ-glutamylcysteine synthetase^52^. Considering our results, a potential attenuation of the Nrf2-Keap1 system may exist in children with ASD. However, besides the Nrf2-Keap1 system, reports on feedback mechanisms from ROS for antioxidants have been rarely elucidated, even in other organs. Miljević et al. describe in patients with schizophrenia an antioxidant negative feedback mechanism that involves the GSH peroxidase^53^. To improve our understanding of these mechanisms, further investigations are necessary.

Based on the presented pathophysiological analysis, our results suggest that the measurement of multiple ROS scavenging activities may be useful as a screening test for ASD in children with an age of 6 years or less. Among these children, an increase in the O_2_^·−^ scavenging activity is the key marker for ASD, and combinations including O_2_^·−^ scavenging activity can serve as highly predictive methods for ASD. Because our matched case–control study enrolled children with a mean age of approximately 4 years, the measurement of ROS scavenging activities may serve as a non-behavioural biomarker in ASD screenings of children before their full development of language communication skills. Severe oxidative stress like hyperbilirubinemia in term neonates has been shown to be associated with increased risk for autism spectrum disorders^54^. Therefore, we are also interested in the ROS scavenging activities in the blood of newborn babies during the neonatal period and whether this will facilitate an early ASD diagnosis. To address this issue, we intend to use the cord blood of the Hamamatsu birth cohort^55^.

There are limitations to the present study. Since the participants were children, the blood sample was not always sufficient to perform all analyses; so, the number of samples differs for each measurement. Since the sample size was limited in this study, we restricted the number of variables in the analysis to three or less to avoid overfitting. The small sample size renders the data preliminary, and a larger study with more participants with ASD is necessary. By increasing the number of cases, it will be possible to create a more accurate evaluation tool. However, recruitment for the current study was limited to a group of children with ASD at one hospital, and these children did not receive psychotropic drugs. Therefore, our data are free from the confounding effects of sex, intelligence, and psychotropic drugs, and thus reflect a certain common metabolic pathology among children with ASD. Moreover, the samples were measured in duplicate. Although triplicate measurements are recommended to exclude spurious results, we could not ensure in the present study that there were enough serum samples for triplicate measurements. Since this study aims to clarify ROS chain reactions and oxidant-antioxidant reactions, we did not specify the reaction sites of these ROS. Moreover, the transmethylation and transsulfuration pathways, including genetic polymorphisms, were not investigated.

In conclusion, our results strongly indicate that oxidative stress in children with ASD is not simply increased but shifted in a complicated pattern involving multiple types of ROS and antioxidants. A decreased protection against hydroxyl radicals may be a fundamental mechanism underlying these changes.

## Methods

### Ethics approval

All procedures were approved by the Ethics Committee of the University of Fukui and the Hamamatsu University School of Medicine, and were conducted in accordance with the Ethical Guidelines for Medical and Health Research Involving Human Subjects of the Ministry of Health, Labour and Welfare of Japan. All participants were given a complete description of the study and provided written informed consent from their parent and/or legal guardian before enrolled.

### Study design, participants, and informed consent

This study was an observational, non-interventional investigation. Using the MULTIS method, we determined an antioxidative profile for each sample combined with the total CoQ10 level, CoQ10 oxidation ratio, α-tocopherol, adiponectin, and 8-OHdG levels. The autistic children were recruited through advocacy groups in cooperation with the Asperger Society Japan (Nagoya, Japan) and the Miyagi Children’s Hospital (Sendai, Japan). By contrast, age-matched TD children were recruited from Hamamatsu and Fukui, Japan by advertisement. All participants underwent a comprehensive assessment of their medical history, and individuals with any neurological or other medical disorders were excluded from the study, e.g., if they had a diagnosis of fragile X syndrome, epileptic seizures, obsessive–compulsive disorder, affective disorders, schizophrenia, any additional psychiatric or neurological diagnoses, liver dysfunction, or unspecified inflammatory complications.

Serum samples of 39 children with ASD and 58 children with TD were analysed. The characteristics of all participants are summarized in Table 1. Since ASD is known to be more common in males, and there was a significant sex difference between the ASD and TD groups, we also conducted a subanalysis restricted to male children, all procedures involving participants were conducted with written informed consent by the study participants or their legal guardians following a complete description of the study. The procedures followed a protocol approved by the Ethics Committee at the University of Fukui before enrolment (approval number #20130089).

### Diagnosis and evaluation of ASD using ADI-R and IQ/DQ

The diagnosis of ASD was made by an experienced child psychiatrist based on the criteria outlined in the Diagnostic and Statistical Manual of Mental Disorders (5th Edition) with clinical interviews and three psychological evaluations: the Japanese version of the Autism Diagnostic Interview-Revised (ADI-R). The ADI-R was conducted by three of the authors (KW, TF, and KJT), who established the research reliability of ADI-R with its developer or independent trainers and are experienced at using the ADI-R Japanese Version clinically^56^. The ADI-R involves a semi-specially formulated structured psychiatric interview with a parent, usually the mother^57^. It is used to confirm the diagnosis and evaluate the core symptoms of ASD, as described in^58^. In brief, ADI-R assessments are based on three separate scores. The ADI-R domain score A quantifies impairment in social interactions (score range 0–32); domain score BV quantifies impairment in communication (score range 0–26); and domain score C quantifies restricted, repetitive, and stereotyped patterns of behaviour and interests (score range 0–16). Higher scores in each domain indicate worse performance. The cut-off scores of domain score A, domain score BV, and domain score C are 10, 8, and 3, respectively. All subjects with autism had scores above the cut-off scores. The ADI-R domain D corresponds to the age of onset criterion for autistic disorder. If the score is 1 or higher, the subject is quite likely to have an age of onset prior to 3 years. All subjects with autism have an age of onset no later than 3 years since no one had an ADI-R domain D scores lower than 1.

To assess the intelligence of participants, we evaluated first the Intelligence quotient (IQ) using the Wechsler Intelligence Scale for Children-Third Edition (WISC-III) for school-age children and second, the developmental quotient (DQ) using the Kyoto Scale of Psychological Development (KSPD) for preschool-age children. A previous report validated that DQ assessments using KSPD seem equivalent to an IQ assessment^59^. The KSPD is one of the most widely used developmental tests in Japan, and DQ has been considered to be equivalent to the IQ of younger ASD children^60^. The KSPD is an individualized face-to-face test administered by experienced psychologists to assess a child’s development in the following three areas: Postural–Motor (P-M; fine and gross motor functions); Cognitive–Adaptive (C-A; non-verbal reasoning or visuospatial perceptions assessed using materials [e.g., blocks, miniature cars, marbles]); and Language–Social (L-S; interpersonal relationships, socialization and verbal abilities)^60^. In each of the three areas, a sum score is converted to a developmental age (DA), and the overall DA is then divided by the child’s chronological age and multiplied by 100 to arrive at the DQ^60^. The validity of DQ on the Mental Development Scale for Infants and Young Children (MDSIYC) has already been tested as an estimate of IQ in a previous study. Correlations were carried out between its DQ with IQ using the Japanese version of the Stanford–Binet in 94 children with autistic disorder, as diagnosed based on Diagnostic and Statistical Manual of Mental Disorders (4th edition; DSM-IV)^61^. With IQ, DQ in the five MDSIYC areas (motor, play, socialization, self-help, and speech) and full-scale DQ (mean of the five-area DQ) had significant correlations. However, unlike IQ, DQ seems to be an index that directly reflects the symptoms of ASD, especially in infants^61^. All enrolled participants were drug-naive and had been free of cholesterol-lowering diets for at least 6 months before this study. The Structured Clinical Interview for DSM-IV (SCID) was also conducted to scrutinize any personal or family history of past or present mental illness.

### Sample collection

Fasting blood samples were collected from all participants between 7:00 and noon by venipuncture in a sitting position using a tourniquet. The blood samples were kept at room temperature for 30 min and centrifuged at 3500×*g* for 10 min in a refrigerated centrifuge. They were then divided into 200-µl aliquots and stored at − 80 °C for subsequent analyses.

### Measurements of free radical scavenging activities in the serum

Multiple free radical scavenging activities were measured by an ESR-based MULTIS method, as described previously^37^. In this method, we evaluated five ROS, namely, ^·^OH, RO^·^, ROO^·^, O_2_^·−^, and ^1^O_2_. Each ROS was produced via in situ illumination with ultraviolet or visible light from an illuminator (RUVF-203SR UV illuminator; Radical Research Inc., Tokyo, Japan). Light sources, illumination times, precursors, and photosensitizers used to produce ROS are summarized in Supplementary Table S1. The ESR spectrometer employed was an RR-X1 unit equipped with 100-kHz field modulation and WIN-RAD operation software (Radical Research Inc.). Typical spectrometer settings were as follows: field modulation width, 0.1 mT; microwave power, 10 mW; field scan width/rate, ± 7.5 mT/2 min; and time constant, 0.1 s. ROS scavenging activities were expressed as the unit equivalent to known pure scavengers; GSH for ^·^OH and ^1^O_2_, SOD for O_2_^·−^, 6-hydroxy-2,5,7,8-tetramethylchromane-2-carboxylic acid for RO^·^, and α-lipoic acid for ROO^·^^37^.

### Measurements of oxidative stress markers, adiponectin, and tocopherols

Laboratory measurements of serum AST, ALT, γ-GT, and CRP levels were determined at SRL Inc. (Tokyo, Japan) using a routine clinical biochemistry automatic analyser. The established oxidative stress markers 8-OHdG, total CoQ10, CoQ10 oxidation rate, adiponectin, and α-tocopherol were measured at the Japan Institute for the Control of Aging (Shizuoka, Japan) according to previously established methods^62,63^.

### Materials

Radical Research Inc. provided 5-(2,2-dimethyl-1,3-propoxy cyclophosphoryl)-5-methyl-1-pyrroline N-oxide (CYPMPO). Hydrogen peroxide, riboflavin, 2,2′-azobis (2-amidinopropane) hydrochloride (AAPH), *tert*-butyl hydroperoxide, dimethyl sulfoxide (DMSO), Rose Bengal, and 4-hydroxy-2,2,6,6-tetramethylpiperidine (4-OH-TEMP) were purchased from Tokyo Chemical Industry (Tokyo, Japan) and used without modification. Buffers and biochemical reagents were obtained from Wako Chemical Co. (Osaka, Japan).

### Statistical analysis

Intergroup comparisons were performed using Student’s unpaired t-test for metric scale or the Mann–Whitney U test for ordinal scale, except for the comparison of the male/female ratio for which the chi-squared test was employed. Subsequently, canonical discriminant analysis models were prepared to estimate the risk of ASD associated with serum ROS scavenging activities. ROS scavenging activities that showed significant differences between the ASD and TD groups were included as variables. We followed standard methods to estimate sample size for multivariate analysis, with approximately ten outcomes needed for each independent variable. Because the sample size was limited, we restricted the number of variables to three or less to avoid overfitting. OR, sensitivity, specificity, LR+, and CC were calculated with the selected ROS and their combinations. The goodness of fit of each model was evaluated using the AUC values.

Statistical analyses were performed using Prism 6 for Mac OS X software (GraphPad Software Inc., La Jolla, CA, USA) in combination with Mac Multivariable Analysis (Esumi, Tokyo, Japan).

## Supplementary information

Supplementary Table S1.

## Data Availability

The datasets generated during and/or analysed during the current study are available in the repository of Tsukuba University of Technology. They are available from the corresponding author upon reasonable request.
