# Supplementary material for: Simultaneous evaluation of antioxidative serum profiles facilitates the diagnostic screening of autism spectrum disorder in under-6-year-old children
Source: Sci Rep. 2020 Nov 26;10:20602. doi: 10.1038/s41598-020-77328-z (PMC7691362; doi:10.1038/s41598-020-77328-z)
Supplement: Supplementary file 1 — Supplementary Table S1. [file 41598_2020_77328_MOESM1_ESM.pdf]

Simultaneous evaluation of antioxidative serum profiles facilitates the diagnostic screening of autism spectrum disorder in under-six-year-old children

Aki Hirayama[1], Keisuke Wakusawa[2], Toru Fujioka[3,4], Keiko Iwata[3,4,5], Noriyoshi Usui[3,4,5,6,7,8], Daisuke Kurita[9], Yosuke Kameno[9], Tomoyasu Wakuda[9], Shu Takagai[4,10], Takaharu Hirai[3,4,11], Takahiro Nara[2], Hiromu Ito[12], Yumiko Nagano[1], Shigeru Oowada[13], Masatsugu Tsujii[14], Kenji J. Tsuchiya[4,15] & Hideo Matsuzaki[3,4,5]*

Supplementary Table S1. Production of multiple reactive oxygen species and specific antioxidants in MULTIS measurements.

ROS	Precursor/ Sensitizer	Irradiation	Spin Trap	Antioxidant Equivalent
$^{\bullet}OH$	H_2O_2 10 mM	UV	CYPMPO	GSH
$RO^{\bullet}$	AAPH 10 mM	UV	CYPMPO	Trolox
$ROO^{\bullet}$	*t*BHP 10 mM	UV	CYPMPO	α-lipoic acid
$O_2^{\bullet-}$	Riboflavin 20 μM	VL	CYPMPO	SOD
$^1O_2$	Rosebengal 200 μM	VL	TMPO	GSH

MULTIS, multiple free radical scavenging activity; ROS, reactive oxygen species; $^{\bullet}OH$, hydroxyl radical; $O_2^{\bullet-}$, superoxide; $RO^{\bullet}$, alkoxyl radical; $ROO^{\bullet}$, peroxyl radical; $^1O_2$, singlet oxygen; AAPH, 2,2'-azobis(2-methyl-propanimidamide) dihydrochloride; *t*BHP, *tert*-butyl hydroperoxide; UV, ultraviolet (300–400 nm); VL, visual light (500–600 nm); CYPMPO, 5-(2,2-dimethyl-1,3-propoxy cyclophosphoryl)-5-methyl-1-pyrroline N-oxide; TMPO, 4-hydroxy-2,2,6,6-tetramethylpiperidine; GSH, glutathione; SOD, superoxide dismutase; Trolox, 6-hydroxy-2,5,7,8-tetramethylchroman-2-carboxylic acid.
